# Loin pain hematuria syndrome (LPHS) induced by pyelonephritis in diabetes mellitus female with brucellosis: a diagnosis of challenge

**DOI:** 10.1097/MS9.0000000000003312

**Published:** 2025-04-22

**Authors:** Mohammad Alsultan, Marwa Kliea, Alaa Aldin Zedan, Kassem Basha

**Affiliations:** a Department of Nephrology, Damascus University- Faculty of medicine, Damascus, Syria; b Department of Internal Medicine, Burjeel Hospital for Advanced Surgery, Dubai, United Arab Emirates

**Keywords:** brucellosis, diabetes mellitus (DM), kidney biopsy, loin pain hematuria syndrome (LPHS), pyelonephritis

## Abstract

Loin pain hematuria syndrome (LPHS) is an extremely rare disease with a prevalence of about 0.012% and is more common in women (approximately 70% of cases). The majority of cases present by the third decade of life, where the chronic loin pain varies in duration (ranging from minutes to a constant ache) and frequency (ranging from once or twice a year to incessant pain). Also, the pain usually accompanies by intermittent microscopic or gross hematuria.

*Dear Editor:*

Loin pain hematuria syndrome (LPHS) is an extremely rare disease with a prevalence of about 0.012% and is more common in women (approximately 70% of cases)^[^1,2^]^. The majority of cases present by the third decade of life, where the chronic loin pain varies in duration (ranging from minutes to a constant ache) and frequency (ranging from once or twice a year to incessant pain)^[^1^]^. Also, the pain usually accompanies by intermittent microscopic or gross hematuria^[^1^]^.

A 40-year-old female with a history of type 1 diabetes mellitus (DM) following a total pancreatic resection for insulinoma at age 20, diabetic neuropathy for 10 years, and diabetic retinopathy for 1 year. She was diagnosed with brucellosis 5 months ago, for which she was treated with doxycycline for 45 days, and has had recent pyelonephritis within the past month. The patient is currently taking insulin, oral iron, and a combination of calcium and vitamin D. She was admitted to the Nephrology Department with a 10-day history of arthralgia, fever, dysuria, oliguria, and pain in both flanks.

Urinalysis showed a turbid appearance, 18 WBCs/HPF (normal: 0–5), 12 RBCs/HPF (normal: 0–3), and positive nitrites, consistent with pyelonephritis. Urine culture showed klebsiella which is sensitive to nitrofurantoin, meropenem, imipenem, and colistin. The patient is covered with meropenem (1 g/3 times/daily). The patient was diagnosed with recurrent brucellosis (antibodies IgM and IgG were positive), and treated with tetracycline, rifampin, and trimethoprim/ sulfamethoxazole. The patient showed significant improvement after a week of treatment for pyelonephritis and brucellosis. Urinary output (UO) improved (without discoloration during rifampicin treatment) and C-reactive protein (CRP) normalized without fever, dysuria, or flank pain.

**HIGHLIGHTS**

- Pyelonephritis can initiate loin pain hematuria syndrome (LPHS).
- LPHS diagnosis needs extensive investigations and long-term follow-up.
- Clinicians should consider LPHS in patients with persistent pain and hematuria.

Ten days after meropenem initiation and symptom improvement, the flank pain recurred which proceeded by one episode of fever (39°C). During the following two days, the patient described dark and red urine (macrohematuria) with gradual UO reduction to be anuric and edematous (pretibial edema grade III); however, serum creatinine (sCr) remained normal. The urinalysis showed 300-350 RBCs/ HPF, where 80% of RBCs were dysmorphic. Complements C3, C4, and ANA were negative. Non-contrast abdominal CT scan and kidney ultrasound were negative for stones and hydronephrosis.

The status suggested glomerulonephritis (GN) (particularly IgA nephropathy) induced by brucellosis or acute tubulointerstitial nephritis induced by pyelonephritis. The kidney biopsy was performed and showed nodular glomerulosclerosis (Kimmelstiel-Wilson lesions), in addition to afferent and efferent arteriosclerosis. Also, tubules showed hyaline casts with acute tubular injury and mild tubulointerstitial fibrosis. The immunofluorescence showed a non-specific linear staining of IgG and negative staining for IgA, IgM, C3, and C1q. Biopsy features consisted with diabetic kidney disease without GN.

UO gradually improved with intravenous (IV) furosemide without a specific or definitive diagnosis. Urine protein/24 hours was 693 mg/day. The patient was discharged after completion of pyelonephritis treatment (for 14 days) and continued on brucellosis treatment for 45 days.

Pyelonephritis recurred a month later, where the urine culture showed the same pathogen (klebsiella) with the same sensitivity. The patient received meropenem and improved within days. After a week, the same episode of flank pain was recurred again and sCr remained normal. Urinalysis showed 200-250 RBCs/ HPF with 80% being dysmorphic, and negative urine culture. Thereafter, UO gradually improved with IV furosemide. Here, the pyelonephritis treatment continued for 21 days and was followed by nitrofurantoin prophylaxis for three months.

The follow-up continued for 3 years. In the first year (2022), flank pain persisted in less severity, which was treated with antidepressants and intermittent analgesics prescriptions. Here, the patient developed three episodes of flank pain with macrohematuria without pyelonephritis or increasing sCr. Also, microhematuria persisted between episodes, which showed glomerular origin. At this year, we suggested the diagnosis of LPHS and continued the patient follow-up (Table 1).

**Table 1:** Timeline of LPHS episodes, Investigations, and treatments.

Time	1st admission	10 d of 1st admission		2nd admission (month later)	7 d of 2nd admission
Symptoms	Flank pain, fever, dysuria, oliguria, arthralgia	One episode of fever followed by flank pain, macrohematuria, anuria, and edema		Pyelonephritis recurred	Same episode of fever followed by flank pain, macrohematuria, anuria, and edema
Investigations	Urinalysis, urine culture, brucella antibodies	Urinalysis (80% dysmorphic RBC). Complements C3, C4, and ANA (negative). Abdominal CT scan and kidney ultrasound (negative). - Kidney biopsy		Urinalysis, urine culture	Urinalysis (80% dysmorphic RBC).
Diagnosis	Pyelonephritis and brucellosis	Diabetic kidney disease with hematuria of glomerular origin		Pyelonephritis	Hematuria of glomerular origin
Treatments	Pyelonephritis: meropenem	Furosemide and previous treatments		Pyelonephritis: meropenem	Continue meropenem followed by nitrofurantoin prophylaxis Furosemide
	Brucellosis: tetracycline, rifampin, and trimethoprim/ sulfamethoxazole				
**Follow-up**					
	**First year (2022)**		**Second year (2023)**	**Third year (2024)**	**Notes:**
					Kidney function remained normal (sCr = 0.9 mg/dL) all over time line. Urine culture showed klebsiella with the same sensitivity in 1st and 2nd admissions. Abdominal CT scan and kidney ultrasound ruled out urinary obstruction and nephrolithiasis.
Symptoms	Flank pain persisted in less severity. Three episodes of flank pain and macrohematuria Microhematuria persisted between episodes		-No flank pain or hematuria	-No flank pain or hematuria	
Investigations	Urinalysis, urine culture (negative).		Urinalysis, urine culture (negative).	Urinalysis, urine culture (negative).	
Treatments	Antidepressants and intermittent analgesics		______	______	
Diagnosis	LPHS		LPHS resolved	LPHS resolved	

In the second year (2023) (Table 1), flank pain and hematuria resolved completely and spontaneously without episodes of anuria or pyelonephritis. Kidney function remained normal (sCr = 0.9 mg/dL) and the urine protein/Cr ratio was 0.17. In the third year (2024) (Table 1), the patient did not describe any episodes of flank pain or hematuria. The final diagnosis consisted of LPHS after the exclusion of glomerular and non-glomerular etiologies (Table 1).

LPHS is a diagnosis of challenge due to the rarity of the disease and many patients with LPHS remain unrecognized for years^[^1,3^]^. Low-grade fever has been reported in many patients and the majority have normal renal function^[^1,2,4^]^. Pain is induced or exacerbated by exercise in approximately one-half of patients and 25–50% of cases spontaneously resolved within 3–5 years^[^2-4^]^. The diagnosis of LPHS required an exclusion of non-glomerular etiologies including nephrolithiasis, urinary obstruction, and pyelonephritis^[^1-3,5^]^. Also, glomerular etiologies should be excluded such as IgA nephropathy, so kidney biopsy is required when the patients show proteinuria (>500 mg/24 h) or increased sCr^[^1,2,4^]^. We ruled out these etiologies, when investigations were ruled out urinary obstruction and nephrolithiasis (by abdominal CT), and GN (by kidney biopsy). Also, pyelonephritis induced the first two episodes, where it was treated appropriately and never recurred. The diagnosis was made several months later, when the patient suffered from continuous pain, three spontaneous episodes of loin pain with macrohematuria, and persistent microhematuria between the episodes.

Here, the diagnostic dilemma in this patient was because of the following:

First, the patient received a previous insufficient treatment for brucellosis, which could follow by GN due to indolent infection. Second, LPHS induced during the pyelonephritis treatment. Third, LPHS occurred after pyelonephritis in the first two episodes. Fourth, all episodes proceeded by high grade fever. To the best of our knowledge, we reported the first case of LPHS induced by pyelonephritis^[^3^]^.

The strategies for LPHS treatment include both pharmacologic and nonpharmacological therapies^[^4,5^]^. The main initial treatment includes analgesics, that commonly include nonsteroidal anti-inflammatory drugs (NSAIDS) combined with muscle relaxants, antidepressants, antiepileptics, or opioids as adjuvant therapy^[^4,6,7^]^. Prasad *et al*^[^3^]^ reported that 30% of LPHS patients had a history of anxiety and depression, so further behavioral therapy and physical medicine would also be applied^[^4^]^. Several patients suffer from severe pain, that may need invasive treatments such as nerve block, denervation, kidney auto-transplantation, and nephrectomy; however, these strategies carry considerable morbidity and costs^[^4,6,7^]^.

In our patient, we started LPHS treatment with analgesics (mainly a combination of paracetamol/codeine phosphate) and antidepressants considering their benefits in treating diabetic neuropathy and the underlying psychiatric basis of chronic pain syndromes^[^3,6,7^]^. Also, opioids and NSAIDS were intermittently prescribed as needed to prevent drug abuse and their side effects.

The diagnosis delay for such LPHS patients could cause several hospital referrals or admissions and increased comorbidities due to chronic pain, in hospital infections, or addiction. So, future efforts should focus on early diagnosis and management of LPHS to reduce these costs and comorbidities.

In summary, LPHS could be triggered by pyelonephritis. Since, there is no definitive utility for LPHS diagnosis and hard to diagnose without a kidney biopsy, imagining investigations, and long-term follow-up, clinicians should consider LPHS in patients with persistent pain and hematuria. LPHS can be exhausted for both patient and doctors, so future studies should be focused on designing criteria for LPHS diagnosis to prevent diagnostic delay and increasing costs of unnecessary investigations.

## Data Availability

The data are available from the corresponding author upon reasonable request.
